# Crystal structure and Hirshfeld surface analysis of bis­(6,7,8,9-tetra­hydro-11*H*-pyrido[2,1-*b*]quinazolin-5-ium) tetra­chlorido­zincate

**DOI:** 10.1107/S2056989021004989

**Published:** 2021-05-14

**Authors:** Akmaljon Tojiboev, Rasul Okmanov, Ulli Englert, Ruimin Wang, Fangfang Pan, Kambarali Turgunov, Bakhodir Tashkhodjaev

**Affiliations:** a Laboratory of Thermal Physics of Multiphase Systems, Arifov Institute of Ion-Plasma and Laser Technologies, Academy of Sciences of Uzbekistan, Durmon yuli str. 33, Tashkent, 100125, Uzbekistan; b S.Yunusov Institute of Chemistry of Plant Substances, Academy of Science of Uzbekistan, Mirzo Ulugbek Str. 77, 100170 Tashkent, Uzbekistan; c Institute of Inorganic Chemistry, RWTH Aachen University, Landoltweg 1, 52056, Aachen, Germany; d College of Chemistry, Key Laboratory of Pesticide and Chemical Biology of Ministry of Education, Hubei International Scientific and Technological Cooperation Base of Pesticide and Green Synthesis, Central China Normal University, Luoyu Road 152, Wuhan, Hubei Province 430079, People’s Republic of China; e Turin Polytechnic University in Tashkent, Kichik Khalka yuli str., 17, 100095 Tashkent, Uzbekistan

**Keywords:** tricyclic quinazoline, inter­molecular inter­actions, Hirshfeld surface, crystal structure

## Abstract

The title compound, (C_12_H_15_N_2_)_2_[ZnCl_4_], is a salt with two symmetrically independent, essentially planar heterocyclic cations and a slightly distorted tetra­hedral chloro­zincate dianion. N—H⋯Cl hydrogen bonds link these ionic constituents into a discrete aggregate, which comprises one formula unit. The effect of hydrogen bonding is reflected in the minor distortions of the [ZnCl_4_]^2−^ moiety: distances between the cation and chlorido ligands engaged in classical hydrogen bonds are significantly longer than the others. Secondary inter­actions comprise C—H⋯π hydrogen bonding and weak π–π stacking. A Hirshfeld surface analysis indicates that the most abundant contacts in packing stem from H⋯H (47.8%) and Cl⋯H/H⋯Cl (29.3%) inter­actions.

## Abstract 2

N—H⋯Cl hydrogen bonds link two di­hydro­quinazolinium cations and a tetra­chlorido zincate dianion into discrete aggregates. Neighbouring (C_12_H_15_N_2_)_2_[ZnCl_4_] units inter­act *via* non-classical C—H⋯π hydrogen bonds and π–π stacking.

## Chemical context   

Tricyclic quinazolines are counted among the most exciting quinazoline alkaloids. Specifically, the alkaloid mackinazoline was isolated from *Mackinlaya sp*. (Johns & Lamberton, 1965 ▸). Tricyclic quinazolines have several different reactive sites and can react with electrophilic and nucleophilic reagents to form various derivatives with potential biological activity (Michael, 2004 ▸). As quinazoline alkaloids are scarcely available from natural sources, multiple methods for their synthesis have been developed (Shakhidoyatov & Elmuradov, 2014 ▸). In the context of these synthetic efforts, reaction inter­mediates similar to the title compound have been studied (Sharma *et al.*, 1993 ▸; Sargaza­kov *et al.*, 1991 ▸; Tozhiboev *et al.*, 2005 ▸). We investigated the crystal structure of bis­(6,7,8,9-tetra­hydro-11*H*-pyrido[2,1-*b*]quinazolin-5-ium) tetra­chlorido­zincate, an inter­mediate in the synthesis of mackinazolinone, using high-resolution diffraction data and Hirshfeld surface analysis.
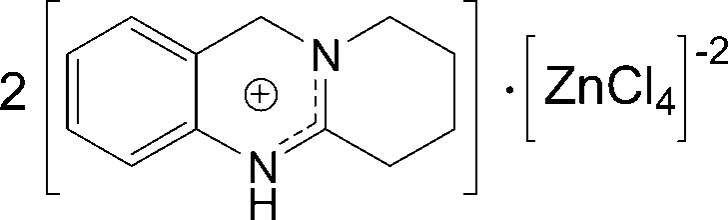


## Structural commentary   

**Figure 1:**
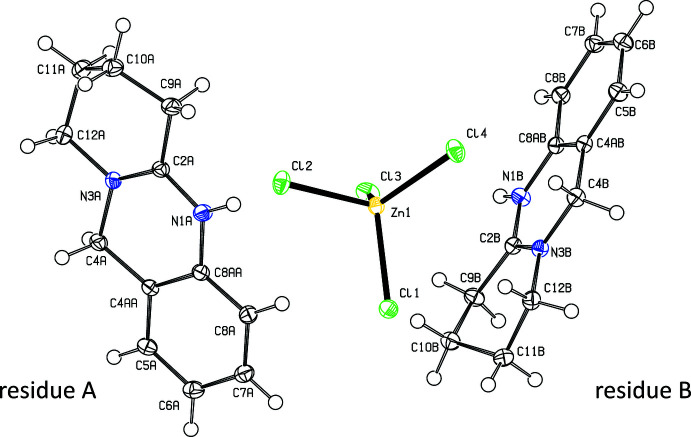
Asymmetric unit of the title compound with the atom-numbering scheme (Spek, 2020 ▸). Displacement ellipsoids for non-hydrogen atoms are drawn at the 50% probability level.

**Figure 2:**
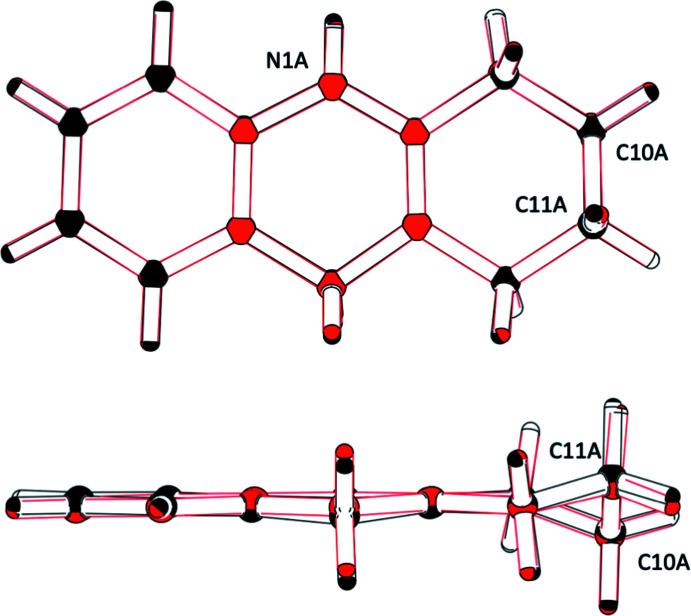
Overlay (Spek, 2020 ▸) of the independent cations in the title compound in the least-squares (top) and most-squares plane (bottom); residue *A* is depicted in black, residue *B* in red.

The title compound crystallizes in the *P*2_1_/*n* space group, with two [C_12_H_15_N_2_]^+^ cations and a [ZnCl_4_]^2−^ counter-anion in the asymmetric unit (Fig. 1 ▸). The benzene and pyrimidine rings in either cation and the attached carbon atoms of the aliphatic ring (C9*A* and C12*A* for residue *A* and C9*B* and C12*B* for residue *B*) are essentially coplanar, with r.m.s. deviations of 0.0437 and 0.0168 Å for mol­ecules *A* and *B*, respectively. The remaining atoms of the third ring are significantly displaced above the opposite faces of these planes with deviations of 0.3877 (12) Å for C10*A* and 0.3831 (11) Å for C11*A* in residue *A* and 0.4705 (11) Å for C10*B* and 0.2495 (11) Å for C11*B* in residue *B*. Fig. 2 ▸ shows that the independent cations are almost superimposable including the conformationally soft aliphatic ring.

The protonation of the ring occurs at the basic heteroatoms of the pyrimidine rings, N1*A* and N1*B*, respectively, and the acquired positive charge is delocalized within the –N–C–N– moiety in the ring, where the N1*A*—C2*A* and N1*B*—C2*B* bonds are only slightly longer than C2*A*—N3*A* and C2*B*—N3*B* (Table 1 ▸). Similar differences were observed in related reported complexes (Sharma *et al.*, 1993 ▸; Turgunov *et al.*, 2003 ▸; Tozhiboev *et al.*, 2005 ▸).

However, these C—N bond lengths are shorter than those in the related tricyclic protonated (PYQAZP: Reck *et al.*, 1974 ▸) and non-protonated (GUCZUZ: Le Gall *et al.*, 1999 ▸; LIZMOX: Zhang *et al.*, 2008 ▸) quinazoline derivatives. In these three compounds, the *sp^3^* character of the carbon atom between the two nitro­gen atoms and the lack of the C=N double bond within the –N–C–N– moiety hampers the delocal­ization of the positive charge within this unit. It is instead delocalized over the –N=CH—C(phenyl­ene) fragment (see Table S1 in the supporting information).

**Figure 3:**
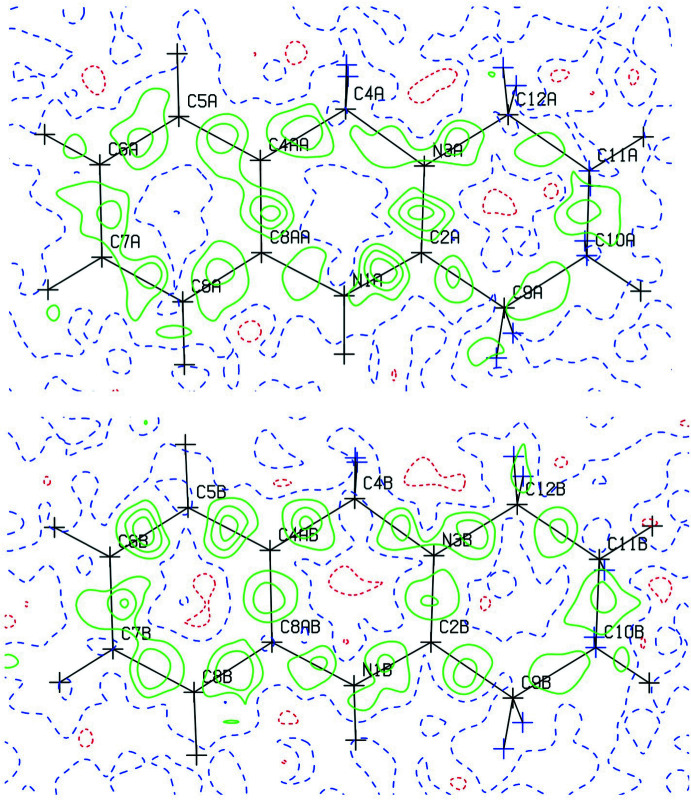
Residual electron density in the planes through C2*A*, C4*A* and C8*AA* (top) and C2*B*, C4*B* and C4*AB* (bottom); contour lines are drawn at 0.2 e Å^−3^. Covalent bonds in the heterocyclic cations clearly show up as local density maxima.

Analysis of the residual electron density (Spek, 2020 ▸) reveals that the covalent bonds in the heterocyclic cations clearly show up as local density maxima (Fig. 3 ▸).

The Zn^II^ centre in the dianion adopts a slightly distorted geometry, with τ^4^ = 0.95 (Yang *et al.*, 2007 ▸). The high resolution (θ_max_ = 109.6°, sin θ/λ = 1.150 Å^−1^, *d* = 0.43 Å) and the very favourable ratio between observations and variables (100:1) in our diffraction data result in small standard uncertainties for atomic coordinates and derived geometric parameters and allow to discuss more subtle details. The most acute angle of 103.33 (11)° within the tetra­chlorido­zincate dianion (Table 1 ▸) is subtended by Cl1 and Cl2. These atoms are associated with the longest Zn—Cl distances, which, in turn, are correlated with the most relevant inter­molecular inter­actions in the structure: Cl1 is involved in the shortest and most linear N—H⋯Cl hydrogen bond (see Table 2 ▸) and represents the most distant ligand in the anion. Cl2 is significantly closer to Zn1 and is engaged in a longer and presumably weaker hydrogen bond. The remaining chlorido ligands are not associated with any classical short contacts. Similar features have been reported for structurally related compounds (Sharma *et al.*, 1993 ▸; Sargaza­kov *et al.*, 1991 ▸; Tozhiboev *et al.*, 2005 ▸; Wang *et al.*, 2017 ▸).

## Supra­molecular features   

**Figure 4:**
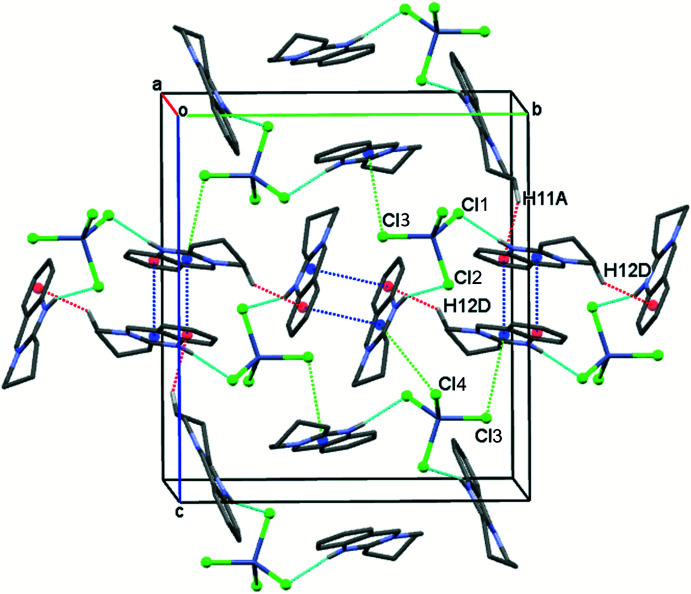
Crystal packing and short contacts in the title compound. Colour code: N—H⋯Cl inter­actions light-blue dashed lines, inter­molecular C—H⋯π contacts red dashed lines, Zn—Cl⋯π contacts green dashed lines, π-π stacking inter­actions dark-blue dashed lines. Centroid for the pyrimidine (*Cg*1, *Cg*7) and benzene rings (*Cg*3, *Cg*9) are shown as blue and red spheres, respectively.

In the crystal structure, the protonated N1*A* and N1*B* nitro­gen atoms in the cations inter­act with the chlorido ligands Cl2 and Cl1, respectively, *via* relatively short N—H⋯Cl bonds and generate a *D*_2_^2^(5) graph-set motif (Bernstein *et al.*, 1995 ▸) (Table 2 ▸ and Fig. 4 ▸).

The crystal packing is further stabilized by inter­molecular C—H⋯π inter­actions (Table 2 ▸) and additional short contacts between Cl3 and the N–C–N segment of the pyrimidine rings. The shortest contact distance occurs between Cl3 and C2*B* [3.5273 (9) Å] and involves an inter­action between the electron-rich equatorial region of the halogen atom and the ring atom attached to two N-atom neighbours, most probably the most electron-deficient atom in the heterocycle. These contacts link anions and cations into a three-dimensional network. Weak π–π stacking inter­actions occur between pyrimidine (*Cg*1, *Cg*7) and benzene (*Cg*3, *Cg*9) rings of anti­parallel pairs of cations and involve contact distances of *Cg*1⋯*Cg*3 (−*x*, −*y*, −*z*) = 3.6225 (5) Å (slippage 0.857 Å) and of *Cg*7⋯*Cg*9 (1 − *x*, −*y*, 1 − *z*) = 3.6246 (7) Å (slippage 0.994 Å).

## Hirshfeld surface analysis   

**Figure 5:**
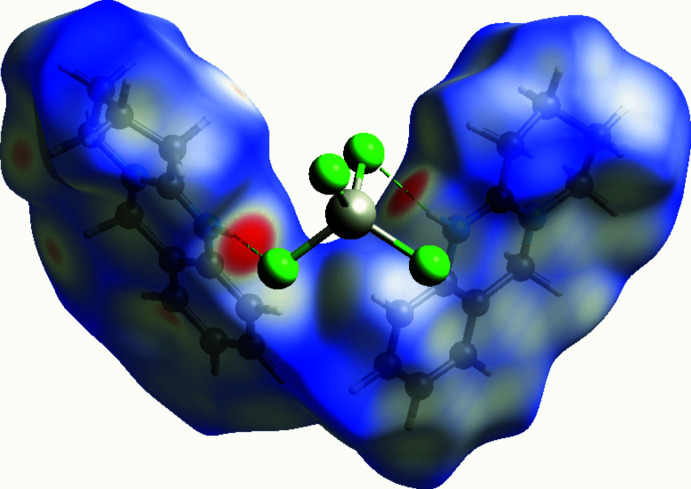
Three-dimensional Hirshfeld surface of the title compound mapped with *d*_norm_.

A Hirshfeld surface (HS) analysis (Spackman & Jayatilaka, 2009 ▸) was carried out using *CrystalExplorer17.5* (Turner *et al.*, 2017 ▸) to visualize inter­actions between the constituents of the title compound. The HS mapped with *d*_norm_ is represented in Fig. 5 ▸. The white surface indicates contacts with distances equal to the sum of van der Waals radii, and the red and blue colours indicate distances shorter or longer than the van der Waals radii, respectively. The bright-red spot near Cl1 indicates its role as a hydrogen-bond donor towards N1.

**Figure 6:**
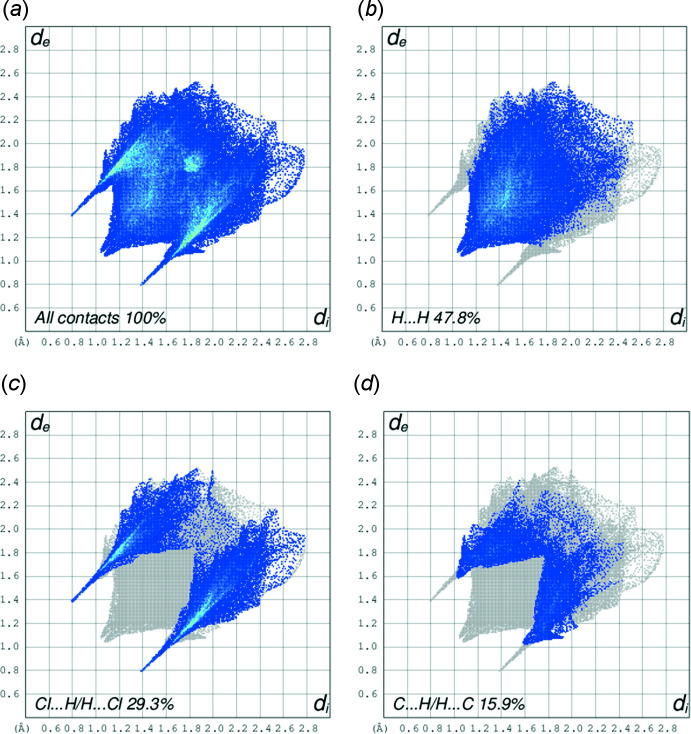
Two-dimensional fingerprint plots for the title compound, showing (*a*) all inter­actions, and decomposed into (*b*) H⋯H, (*c*) Cl⋯H/H⋯Cl, (*d*) C⋯H/H⋯C inter­actions. Values for *d*_i_ and *d*_e_ represent the closest inter­nal and external distances (in Å) from given points on the Hirshfeld surface.

The classical N—H⋯Cl hydrogen bonds correspond to Cl⋯H/H⋯Cl contacts (29.3% contribution) in Fig. 6 ▸ *c* and show up as a pair of spikes. The most abundant contributions to the Hirshfeld surface arise from H⋯H contacts at 47.8%. Cl⋯H/H⋯Cl and C⋯H/H⋯C inter­actions follow with contributions of 29.3% and 15.9%, respectively (Fig. 6 ▸). Minor contributors are due to C⋯N/N⋯C (2.2%), N⋯H/H⋯N (2.0%), C⋯C (1.9%), C⋯Cl/Cl⋯C (0.4%), N⋯Cl/Cl⋯N (0.3%) and Zn⋯H/H⋯Zn (0.3%) contacts.

## Database survey   

A search in the Cambridge Structural Database (CSD, version 5.41, including the update of January 2020; Groom *et al.*, 2016 ▸) confirmed that four related compounds had been structurally characterized in which similar cations inter­act with [ZnCl_4_]^2−^ anions. They are associated with refcodes PODLUP (Sharma *et al.*, 1993 ▸), PODLUP01 (Sargaza­kov *et al.*, 1991 ▸) and SECFAI and SECFAI01 (Tozhiboev *et al.*, 2005 ▸). An additional match for a similar cation inter­acting with a Cl^−^ anion was identified: EYUHEL (Turgunov *et al.*, 2003 ▸) and PYQAZP (Reck *et al.*, 1974 ▸).

## Synthesis and crystallization   

**Figure 7:**
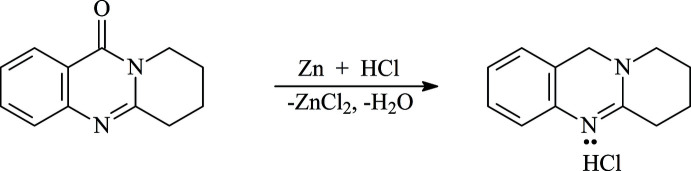
Synthesis scheme for 2,3-tetra­methyl­ene-3,4-di­hydro­quinazoline hydro­chloride.

3 g (0.015 mol) of 2,3-tetra­methyl­enquinazoline-4-one (Fig. 7 ▸) were placed in a 300 mL flat-bottom flask equipped with a magnetic stirrer and a reflux condenser. 72 mL of hydro­chloric acid (15%) were added under stirring. 12 g of Zn powder were added in small portions over a period of 1 h, and the mixture was heated in a water bath for 4 h. The hot reaction mixture was filtered and the filtrate was left to precipitate overnight. The precipitate corresponding to 2,3-tetra­methyl­enquinazoline hydro­chloride was removed by filtration (Fig. 7 ▸). Colourless single crystals of the title compound were obtained by slow evaporation of the resulting filtrate at room temperature.

## Refinement   

Crystal data, data collection and structure refinement details are summarized in Table 3 ▸. H atoms attached to C were positioned geometrically, with C—H = 0.95 Å (for aromatic) or C—H = 0.99 Å (for methyl­ene H atoms), and were refined with *U*_iso_(H) = 1.2*U*_eq_(C). H atoms bonded to nitro­gen were located in a difference-Fourier map, and their positional and isotropic displacement parameters were freely refined.

## Supplementary Material

Crystal structure: contains datablock(s) I. DOI: 10.1107/S2056989021004989/jq2006sup1.cif

Structure factors: contains datablock(s) I. DOI: 10.1107/S2056989021004989/jq2006Isup3.hkl

Supporting information file. DOI: 10.1107/S2056989021004989/jq2006sup4.docx

CCDC reference: 2082994

Additional supporting information:  crystallographic information; 3D view; checkCIF report

## Figures and Tables

**Table 1:** Selected geometric parameters (Å, °)

Zn1—Cl4	2.2484 (3)	N1*A*—C2*A*	1.3373 (11)
Zn1—Cl3	2.2664 (4)	N1*B*—C2*B*	1.3317 (10)
Zn1—Cl2	2.2868 (4)	C2*A*—N3*A*	1.3102 (10)
Zn1—Cl1	2.3019 (3)	C2*B*—N3*B*	1.3114 (9)
			
Cl4—Zn1—Cl3	111.219 (10)	Cl4—Zn1—Cl1	109.994 (13)
Cl4—Zn1—Cl2	115.057 (11)	Cl3—Zn1—Cl1	110.340 (12)
Cl3—Zn1—Cl2	106.573 (10)	Cl2—Zn1—Cl1	103.331 (11)

**Table 2:** Hydrogen-bond geometry (Å, °) *Cg*3 and *Cg*9 are the centroids of the C5*A*–C8*A*/C4*AA*/C8*AA* and C5*B*–C8*B*/C4*AB*/C8*AB* rings, respectively.

*D*—H⋯*A*	*D*—H	H⋯*A*	*D*⋯*A*	*D*—H⋯*A*
N1*A*—H1*A*⋯Cl2	0.89 (2)	2.44 (2)	3.2659 (8)	155.9 (19)
N1*B*—H1*B*⋯Cl1^i^	0.83 (2)	2.352 (19)	3.1661 (7)	166.6 (18)
C11*A*—H11*A*⋯*Cg*9^ii^	0.99	2.67	3.5718 (10)	151
C12*B*—H12*D*⋯*Cg*3^iii^	0.99	2.57	3.4002 (10)	142

Symmetry codes: (i) -x+{\script{1\over 2}}, y-{\script{1\over 2}}, -z+{\script{1\over 2}}; (ii) -x+1, -y, -z; (iii) x+{\script{1\over 2}}, -y+{\script{1\over 2}}, z+{\script{1\over 2}}.

**Table 3:** Experimental details

Crystal data	
Chemical formula	(C_12_H_15_N_2_)_2_[ZnCl_4_]
*M*_r_	581.69
Crystal system, space group	Monoclinic, *P*2_1_/*n*
Temperature (K)	100
*a*, *b*, *c* (Å)	9.2910 (13), 15.682 (2), 17.275 (2)
β (°)	95.642 (2)
*V* (Å^3^)	2504.7 (6)
*Z*	4
Radiation type	Mo *K*α
μ (mm^−1^)	1.43
Crystal size (mm)	0.30 × 0.25 × 0.23

Data collection	
Diffractometer	Bruker D8 gonimeter with *APEX* CCD detector
Absorption correction	Multi-scan (*SADABS*; Bruker, 2008 ▸)
*T*_min_, *T*_max_	0.634, 0.751
No. of measured, independent and observed [*I* > 2σ(*I*)] reflections	170944, 31478, 21664
*R*_int_	0.071
(sin θ/λ)_max_ (Å^−1^)	1.150

Refinement	
*R*[*F* ^2^ > 2σ(*F* ^2^)], *wR*(*F* ^2^), *S*	0.047, 0.124, 1.04
No. of reflections	31478
No. of parameters	306
H-atom treatment	H atoms treated by a mixture of independent and constrained refinement
Δρ_max_, Δρ_min_ (e Å^−3^)	1.29, −0.54

Computer programs: *APEX2* (Bruker, 2008 ▸), and *SAINT* (Bruker, 2008 ▸), *SHELXS97* (Sheldrick, 2008 ▸), *SHELXL2018/3* (Sheldrick, 2015 ▸), *PLATON* (Spek, 2020 ▸), *publCIF* (Westrip, 2010 ▸).

## supplementary crystallographic information

### Crystal data

**Table:**

(C_12_H_15_N_2_)_2_[ZnCl_4_]	*F*(000) = 1200
*M**_r_* = 581.69	*D*_x_ = 1.543 Mg m^−^^3^
Monoclinic, *P*2_1_/*n*	Mo *K*α radiation, λ = 0.71073 Å
*a* = 9.2910 (13) Å	Cell parameters from 9853 reflections
*b* = 15.682 (2) Å	θ = 2.4–53.5°
*c* = 17.275 (2) Å	µ = 1.43 mm^−^^1^
β = 95.642 (2)°	*T* = 100 K
*V* = 2504.7 (6) Å^3^	Block, colourless
*Z* = 4	0.30 × 0.25 × 0.23 mm

### Data collection

**Table:**

Bruker D8 gonimeter with APEX CCD detector diffractometer	31478 independent reflections
Radiation source: Incoatec microsource	21664 reflections with *I* > 2σ(*I*)
Multilayer optics monochromator	*R*_int_ = 0.071
ω scans	θ_max_ = 54.8°, θ_min_ = 1.8°
Absorption correction: multi-scan (SADABS; Bruker, 2008)	*h* = −21→20
*T*_min_ = 0.634, *T*_max_ = 0.751	*k* = −35→35
170944 measured reflections	*l* = −39→37

### Refinement

**Table:**

Refinement on *F*^2^	0 restraints
Least-squares matrix: full	Hydrogen site location: mixed
*R*[*F*^2^ > 2σ(*F*^2^)] = 0.047	H atoms treated by a mixture of independent and constrained refinement
*wR*(*F*^2^) = 0.124	*w* = 1/[σ^2^(*F*_o_^2^) + (0.0465*P*)^2^ + 0.1208*P*] where *P* = (*F*_o_^2^ + 2*F*_c_^2^)/3
*S* = 1.04	(Δ/σ)_max_ = 0.001
31478 reflections	Δρ_max_ = 1.29 e Å^−^^3^
306 parameters	Δρ_min_ = −0.54 e Å^−^^3^

### Special details

**Table:**

Geometry. All esds (except the esd in the dihedral angle between two l.s. planes) are estimated using the full covariance matrix. The cell esds are taken into account individually in the estimation of esds in distances, angles and torsion angles; correlations between esds in cell parameters are only used when they are defined by crystal symmetry. An approximate (isotropic) treatment of cell esds is used for estimating esds involving l.s. planes.

### Fractional atomic coordinates and isotropic or equivalent isotropic displacement parameters (Å^2^)

**Table:**

	*x*	*y*	*z*	*U*_iso_*/*U*_eq_	
Zn1	0.40341 (2)	0.23046 (2)	0.17943 (2)	0.01331 (2)	
Cl1	0.22695 (2)	0.31275 (2)	0.22716 (2)	0.01745 (3)	
N1A	0.16789 (8)	0.13353 (5)	−0.01919 (5)	0.01731 (11)	
H1A	0.233 (2)	0.1607 (14)	0.0126 (12)	0.033 (5)*	
N1B	0.38700 (8)	−0.02546 (4)	0.36997 (4)	0.01498 (9)	
H1B	0.344 (2)	−0.0665 (13)	0.3479 (11)	0.027 (5)*	
Cl2	0.40142 (3)	0.27504 (2)	0.05329 (2)	0.01999 (4)	
C2A	0.20841 (8)	0.09703 (5)	−0.08356 (5)	0.01372 (9)	
C2B	0.31376 (8)	0.04719 (5)	0.37362 (4)	0.01304 (9)	
Cl3	0.33617 (3)	0.09149 (2)	0.17498 (2)	0.01962 (4)	
N3A	0.11462 (7)	0.05870 (4)	−0.13348 (4)	0.01329 (8)	
N3B	0.37680 (8)	0.11694 (4)	0.40176 (4)	0.01312 (8)	
Cl4	0.61489 (3)	0.24837 (2)	0.25278 (2)	0.02103 (4)	
C4A	−0.04199 (9)	0.06077 (6)	−0.12794 (5)	0.01591 (11)	
H4AA	−0.081466	0.002500	−0.136382	0.019*	
H4AB	−0.088035	0.097703	−0.169781	0.019*	
C4B	0.53321 (9)	0.12358 (5)	0.42526 (5)	0.01502 (10)	
H4BA	0.548031	0.145493	0.479203	0.018*	
H4BB	0.576081	0.165215	0.391071	0.018*	
C4AA	−0.08071 (8)	0.09313 (5)	−0.05096 (4)	0.01320 (9)	
C4AB	0.61022 (8)	0.04003 (5)	0.42109 (4)	0.01289 (9)	
C5A	−0.22295 (9)	0.09044 (5)	−0.03207 (5)	0.01629 (11)	
H5AA	−0.295685	0.064510	−0.066819	0.020*	
C5B	0.75850 (9)	0.03353 (5)	0.44269 (5)	0.01662 (11)	
H5BA	0.811468	0.082125	0.462049	0.020*	
C6A	−0.25917 (10)	0.12560 (6)	0.03755 (6)	0.01813 (12)	
H6AA	−0.356791	0.124795	0.049536	0.022*	
C6B	0.82907 (10)	−0.04420 (6)	0.43590 (5)	0.01798 (12)	
H6BA	0.929998	−0.048320	0.450822	0.022*	
C7A	−0.15242 (10)	0.16192 (6)	0.08958 (5)	0.01727 (11)	
H7AA	−0.177180	0.185134	0.137308	0.021*	
C7B	0.75264 (10)	−0.11583 (5)	0.40740 (5)	0.01668 (11)	
H7BA	0.801428	−0.168535	0.402655	0.020*	
C8A	−0.00940 (10)	0.16428 (5)	0.07178 (5)	0.01643 (11)	
H8AA	0.063845	0.188750	0.107176	0.020*	
C8B	0.60526 (9)	−0.11006 (5)	0.38597 (5)	0.01506 (10)	
H8BA	0.552357	−0.158681	0.366592	0.018*	
C8AA	0.02476 (8)	0.13018 (5)	0.00118 (5)	0.01397 (10)	
C8AB	0.53532 (8)	−0.03207 (5)	0.39314 (4)	0.01263 (9)	
C9A	0.36640 (9)	0.09994 (6)	−0.09460 (6)	0.01879 (13)	
H9AA	0.417435	0.055347	−0.061905	0.023*	
H9AB	0.406030	0.155936	−0.076687	0.023*	
C9B	0.15598 (9)	0.04381 (6)	0.34654 (5)	0.01737 (11)	
H9BA	0.104080	0.016581	0.387400	0.021*	
H9BB	0.141921	0.007614	0.299452	0.021*	
C10A	0.39598 (10)	0.08639 (6)	−0.17905 (6)	0.01922 (13)	
H10A	0.364050	0.136876	−0.210599	0.023*	
H10B	0.500890	0.078255	−0.182272	0.023*	
C10B	0.08950 (10)	0.13131 (6)	0.32782 (5)	0.01831 (12)	
H10C	0.120478	0.152492	0.278085	0.022*	
H10D	−0.017322	0.126944	0.322279	0.022*	
C11A	0.31331 (10)	0.00783 (6)	−0.20972 (5)	0.01870 (12)	
H11A	0.334131	−0.003545	−0.263874	0.022*	
H11B	0.344925	−0.042305	−0.177597	0.022*	
C11B	0.13845 (11)	0.19299 (6)	0.39317 (6)	0.01927 (13)	
H11C	0.096932	0.250072	0.380703	0.023*	
H11D	0.101903	0.173279	0.442051	0.023*	
C12A	0.15251 (10)	0.02154 (6)	−0.20731 (5)	0.01692 (11)	
H12A	0.116845	0.059773	−0.250607	0.020*	
H12B	0.102340	−0.033882	−0.215627	0.020*	
C12B	0.30183 (10)	0.19950 (5)	0.40475 (5)	0.01622 (11)	
H12C	0.334665	0.237291	0.364075	0.019*	
H12D	0.330136	0.226467	0.455799	0.019*	

### Atomic displacement parameters (Å^2^)

**Table:**

	*U*^11^	*U*^22^	*U*^33^	*U*^12^	*U*^13^	*U*^23^
Zn1	0.01365 (3)	0.01277 (3)	0.01329 (3)	−0.00112 (2)	0.00014 (2)	−0.00022 (2)
Cl1	0.02079 (8)	0.01451 (6)	0.01701 (7)	0.00525 (6)	0.00166 (5)	0.00210 (5)
N1A	0.0117 (2)	0.0220 (3)	0.0181 (3)	−0.00204 (19)	0.00103 (18)	−0.0070 (2)
N1B	0.0141 (2)	0.01106 (19)	0.0192 (3)	−0.00093 (16)	−0.00121 (18)	−0.00287 (17)
Cl2	0.02268 (9)	0.02343 (9)	0.01374 (7)	−0.00702 (7)	0.00123 (6)	0.00172 (6)
C2A	0.0117 (2)	0.0145 (2)	0.0149 (2)	−0.00067 (18)	0.00108 (17)	−0.00124 (18)
C2B	0.0142 (2)	0.0115 (2)	0.0135 (2)	−0.00064 (17)	0.00143 (17)	−0.00060 (16)
Cl3	0.01743 (8)	0.01217 (6)	0.02897 (10)	−0.00094 (5)	0.00082 (6)	−0.00157 (6)
N3A	0.0132 (2)	0.0139 (2)	0.0127 (2)	−0.00007 (16)	0.00030 (15)	−0.00098 (15)
N3B	0.0150 (2)	0.01093 (18)	0.0134 (2)	−0.00029 (16)	0.00118 (16)	−0.00126 (15)
Cl4	0.01708 (8)	0.02447 (9)	0.02043 (8)	−0.00490 (7)	−0.00384 (6)	0.00143 (6)
C4A	0.0116 (2)	0.0189 (3)	0.0168 (3)	−0.0002 (2)	−0.00079 (19)	−0.0033 (2)
C4B	0.0157 (3)	0.0120 (2)	0.0171 (3)	−0.00189 (19)	0.0005 (2)	−0.00154 (18)
C4AA	0.0116 (2)	0.0128 (2)	0.0150 (2)	0.00039 (17)	0.00005 (17)	−0.00033 (17)
C4AB	0.0142 (2)	0.0118 (2)	0.0128 (2)	−0.00191 (18)	0.00138 (17)	−0.00057 (16)
C5A	0.0123 (2)	0.0165 (3)	0.0201 (3)	−0.0004 (2)	0.0017 (2)	0.0008 (2)
C5B	0.0146 (3)	0.0158 (3)	0.0192 (3)	−0.0023 (2)	0.0005 (2)	−0.0004 (2)
C6A	0.0158 (3)	0.0180 (3)	0.0212 (3)	0.0010 (2)	0.0052 (2)	0.0023 (2)
C6B	0.0142 (3)	0.0190 (3)	0.0206 (3)	0.0000 (2)	0.0011 (2)	0.0011 (2)
C7A	0.0192 (3)	0.0164 (3)	0.0169 (3)	0.0027 (2)	0.0048 (2)	0.0008 (2)
C7B	0.0165 (3)	0.0154 (3)	0.0182 (3)	0.0022 (2)	0.0023 (2)	0.0003 (2)
C8A	0.0177 (3)	0.0159 (3)	0.0156 (3)	0.0021 (2)	0.0011 (2)	−0.0019 (2)
C8B	0.0161 (3)	0.0125 (2)	0.0165 (3)	0.00052 (19)	0.0011 (2)	−0.00113 (18)
C8AA	0.0123 (2)	0.0140 (2)	0.0154 (2)	0.00074 (18)	0.00041 (18)	−0.00140 (18)
C8AB	0.0134 (2)	0.0115 (2)	0.0130 (2)	−0.00062 (17)	0.00100 (17)	−0.00091 (16)
C9A	0.0123 (3)	0.0234 (3)	0.0208 (3)	−0.0012 (2)	0.0024 (2)	−0.0026 (3)
C9B	0.0138 (3)	0.0171 (3)	0.0209 (3)	−0.0010 (2)	0.0007 (2)	−0.0004 (2)
C10A	0.0168 (3)	0.0210 (3)	0.0207 (3)	−0.0002 (2)	0.0060 (2)	0.0014 (2)
C10B	0.0165 (3)	0.0192 (3)	0.0190 (3)	0.0028 (2)	0.0008 (2)	−0.0008 (2)
C11A	0.0203 (3)	0.0198 (3)	0.0167 (3)	0.0020 (2)	0.0057 (2)	0.0001 (2)
C11B	0.0195 (3)	0.0196 (3)	0.0190 (3)	0.0047 (2)	0.0032 (2)	−0.0025 (2)
C12A	0.0190 (3)	0.0186 (3)	0.0130 (2)	−0.0003 (2)	0.0012 (2)	−0.0017 (2)
C12B	0.0206 (3)	0.0122 (2)	0.0158 (3)	0.0021 (2)	0.0009 (2)	−0.00163 (19)

### Geometric parameters (Å, º)

**Table:**

Zn1—Cl4	2.2484 (3)	C6A—H6AA	0.9500
Zn1—Cl3	2.2664 (4)	C6B—C7B	1.3928 (13)
Zn1—Cl2	2.2868 (4)	C6B—H6BA	0.9500
Zn1—Cl1	2.3019 (3)	C7A—C8A	1.3936 (13)
N1A—C2A	1.3373 (11)	C7A—H7AA	0.9500
N1A—C8AA	1.4096 (11)	C7B—C8B	1.3858 (12)
N1A—H1A	0.89 (2)	C7B—H7BA	0.9500
N1B—C2B	1.3317 (10)	C8A—C8AA	1.3965 (11)
N1B—C8AB	1.4005 (10)	C8A—H8AA	0.9500
N1B—H1B	0.83 (2)	C8B—C8AB	1.3962 (11)
C2A—N3A	1.3102 (10)	C8B—H8BA	0.9500
C2A—C9A	1.4994 (12)	C9A—C10A	1.5257 (14)
C2B—N3B	1.3114 (9)	C9A—H9AA	0.9900
C2B—C9B	1.4952 (12)	C9A—H9AB	0.9900
N3A—C4A	1.4680 (11)	C9B—C10B	1.5262 (13)
N3A—C12A	1.4759 (11)	C9B—H9BA	0.9900
N3B—C12B	1.4735 (10)	C9B—H9BB	0.9900
N3B—C4B	1.4735 (11)	C10A—C11A	1.5193 (14)
C4A—C4AA	1.4998 (11)	C10A—H10A	0.9900
C4A—H4AA	0.9900	C10A—H10B	0.9900
C4A—H4AB	0.9900	C10B—C11B	1.5218 (13)
C4B—C4AB	1.4981 (11)	C10B—H10C	0.9900
C4B—H4BA	0.9900	C10B—H10D	0.9900
C4B—H4BB	0.9900	C11A—C12A	1.5140 (13)
C4AA—C8AA	1.3912 (11)	C11A—H11A	0.9900
C4AA—C5A	1.3927 (11)	C11A—H11B	0.9900
C4AB—C8AB	1.3891 (10)	C11B—C12B	1.5149 (14)
C4AB—C5B	1.3951 (12)	C11B—H11C	0.9900
C5A—C6A	1.3941 (13)	C11B—H11D	0.9900
C5A—H5AA	0.9500	C12A—H12A	0.9900
C5B—C6B	1.3944 (13)	C12A—H12B	0.9900
C5B—H5BA	0.9500	C12B—H12C	0.9900
C6A—C7A	1.3930 (14)	C12B—H12D	0.9900
			
Cl4—Zn1—Cl3	111.219 (10)	C7A—C8A—C8AA	119.08 (8)
Cl4—Zn1—Cl2	115.057 (11)	C7A—C8A—H8AA	120.5
Cl3—Zn1—Cl2	106.573 (10)	C8AA—C8A—H8AA	120.5
Cl4—Zn1—Cl1	109.994 (13)	C7B—C8B—C8AB	119.32 (7)
Cl3—Zn1—Cl1	110.340 (12)	C7B—C8B—H8BA	120.3
Cl2—Zn1—Cl1	103.331 (11)	C8AB—C8B—H8BA	120.3
C2A—N1A—C8AA	122.76 (7)	C4AA—C8AA—C8A	121.28 (7)
C2A—N1A—H1A	119.3 (14)	C4AA—C8AA—N1A	118.41 (7)
C8AA—N1A—H1A	117.9 (14)	C8A—C8AA—N1A	120.31 (7)
C2B—N1B—C8AB	122.87 (6)	C4AB—C8AB—C8B	121.48 (7)
C2B—N1B—H1B	117.3 (14)	C4AB—C8AB—N1B	118.97 (7)
C8AB—N1B—H1B	119.4 (14)	C8B—C8AB—N1B	119.53 (7)
N3A—C2A—N1A	121.36 (7)	C2A—C9A—C10A	112.83 (7)
N3A—C2A—C9A	121.75 (7)	C2A—C9A—H9AA	109.0
N1A—C2A—C9A	116.87 (7)	C10A—C9A—H9AA	109.0
N3B—C2B—N1B	121.29 (7)	C2A—C9A—H9AB	109.0
N3B—C2B—C9B	122.27 (7)	C10A—C9A—H9AB	109.0
N1B—C2B—C9B	116.41 (7)	H9AA—C9A—H9AB	107.8
C2A—N3A—C4A	123.12 (7)	C2B—C9B—C10B	113.48 (7)
C2A—N3A—C12A	123.38 (7)	C2B—C9B—H9BA	108.9
C4A—N3A—C12A	112.78 (6)	C10B—C9B—H9BA	108.9
C2B—N3B—C12B	123.39 (7)	C2B—C9B—H9BB	108.9
C2B—N3B—C4B	123.61 (7)	C10B—C9B—H9BB	108.9
C12B—N3B—C4B	112.65 (6)	H9BA—C9B—H9BB	107.7
N3A—C4A—C4AA	113.01 (6)	C11A—C10A—C9A	108.29 (7)
N3A—C4A—H4AA	109.0	C11A—C10A—H10A	110.0
C4AA—C4A—H4AA	109.0	C9A—C10A—H10A	110.0
N3A—C4A—H4AB	109.0	C11A—C10A—H10B	110.0
C4AA—C4A—H4AB	109.0	C9A—C10A—H10B	110.0
H4AA—C4A—H4AB	107.8	H10A—C10A—H10B	108.4
N3B—C4B—C4AB	112.81 (6)	C11B—C10B—C9B	109.21 (7)
N3B—C4B—H4BA	109.0	C11B—C10B—H10C	109.8
C4AB—C4B—H4BA	109.0	C9B—C10B—H10C	109.8
N3B—C4B—H4BB	109.0	C11B—C10B—H10D	109.8
C4AB—C4B—H4BB	109.0	C9B—C10B—H10D	109.8
H4BA—C4B—H4BB	107.8	H10C—C10B—H10D	108.3
C8AA—C4AA—C5A	119.02 (7)	C12A—C11A—C10A	109.98 (7)
C8AA—C4AA—C4A	120.02 (7)	C12A—C11A—H11A	109.7
C5A—C4AA—C4A	120.88 (7)	C10A—C11A—H11A	109.7
C8AB—C4AB—C5B	118.77 (7)	C12A—C11A—H11B	109.7
C8AB—C4AB—C4B	120.22 (7)	C10A—C11A—H11B	109.7
C5B—C4AB—C4B	120.99 (7)	H11A—C11A—H11B	108.2
C4AA—C5A—C6A	120.35 (8)	C12B—C11B—C10B	111.30 (7)
C4AA—C5A—H5AA	119.8	C12B—C11B—H11C	109.4
C6A—C5A—H5AA	119.8	C10B—C11B—H11C	109.4
C6B—C5B—C4AB	120.10 (8)	C12B—C11B—H11D	109.4
C6B—C5B—H5BA	120.0	C10B—C11B—H11D	109.4
C4AB—C5B—H5BA	120.0	H11C—C11B—H11D	108.0
C7A—C6A—C5A	120.09 (8)	N3A—C12A—C11A	113.56 (7)
C7A—C6A—H6AA	120.0	N3A—C12A—H12A	108.9
C5A—C6A—H6AA	120.0	C11A—C12A—H12A	108.9
C7B—C6B—C5B	120.48 (8)	N3A—C12A—H12B	108.9
C7B—C6B—H6BA	119.8	C11A—C12A—H12B	108.9
C5B—C6B—H6BA	119.8	H12A—C12A—H12B	107.7
C6A—C7A—C8A	120.15 (8)	N3B—C12B—C11B	114.03 (7)
C6A—C7A—H7AA	119.9	N3B—C12B—H12C	108.7
C8A—C7A—H7AA	119.9	C11B—C12B—H12C	108.7
C8B—C7B—C6B	119.85 (8)	N3B—C12B—H12D	108.7
C8B—C7B—H7BA	120.1	C11B—C12B—H12D	108.7
C6B—C7B—H7BA	120.1	H12C—C12B—H12D	107.6
			
C8AA—N1A—C2A—N3A	2.92 (13)	C5A—C4AA—C8AA—C8A	0.18 (12)
C8AA—N1A—C2A—C9A	−175.40 (8)	C4A—C4AA—C8AA—C8A	177.12 (8)
C8AB—N1B—C2B—N3B	−2.13 (12)	C5A—C4AA—C8AA—N1A	−179.12 (8)
C8AB—N1B—C2B—C9B	179.87 (7)	C4A—C4AA—C8AA—N1A	−2.18 (11)
N1A—C2A—N3A—C4A	7.04 (12)	C7A—C8A—C8AA—C4AA	−0.81 (12)
C9A—C2A—N3A—C4A	−174.72 (8)	C7A—C8A—C8AA—N1A	178.47 (8)
N1A—C2A—N3A—C12A	176.62 (8)	C2A—N1A—C8AA—C4AA	−5.18 (13)
C9A—C2A—N3A—C12A	−5.15 (12)	C2A—N1A—C8AA—C8A	175.52 (8)
N1B—C2B—N3B—C12B	177.97 (7)	C5B—C4AB—C8AB—C8B	−0.34 (12)
C9B—C2B—N3B—C12B	−4.15 (12)	C4B—C4AB—C8AB—C8B	177.67 (7)
N1B—C2B—N3B—C4B	5.30 (12)	C5B—C4AB—C8AB—N1B	−178.78 (7)
C9B—C2B—N3B—C4B	−176.82 (7)	C4B—C4AB—C8AB—N1B	−0.77 (11)
C2A—N3A—C4A—C4AA	−13.21 (11)	C7B—C8B—C8AB—C4AB	0.19 (12)
C12A—N3A—C4A—C4AA	176.22 (7)	C7B—C8B—C8AB—N1B	178.62 (8)
C2B—N3B—C4B—C4AB	−5.72 (11)	C2B—N1B—C8AB—C4AB	−0.11 (12)
C12B—N3B—C4B—C4AB	−179.08 (7)	C2B—N1B—C8AB—C8B	−178.59 (8)
N3A—C4A—C4AA—C8AA	10.43 (11)	N3A—C2A—C9A—C10A	21.96 (12)
N3A—C4A—C4AA—C5A	−172.68 (7)	N1A—C2A—C9A—C10A	−159.73 (8)
N3B—C4B—C4AB—C8AB	3.35 (10)	N3B—C2B—C9B—C10B	20.32 (12)
N3B—C4B—C4AB—C5B	−178.68 (7)	N1B—C2B—C9B—C10B	−161.70 (8)
C8AA—C4AA—C5A—C6A	0.99 (12)	C2A—C9A—C10A—C11A	−49.37 (10)
C4A—C4AA—C5A—C6A	−175.93 (8)	C2B—C9B—C10B—C11B	−46.34 (10)
C8AB—C4AB—C5B—C6B	0.16 (12)	C9A—C10A—C11A—C12A	61.61 (10)
C4B—C4AB—C5B—C6B	−177.84 (8)	C9B—C10B—C11B—C12B	57.99 (10)
C4AA—C5A—C6A—C7A	−1.52 (13)	C2A—N3A—C12A—C11A	17.27 (11)
C4AB—C5B—C6B—C7B	0.17 (14)	C4A—N3A—C12A—C11A	−172.19 (7)
C5A—C6A—C7A—C8A	0.87 (13)	C10A—C11A—C12A—N3A	−45.61 (10)
C5B—C6B—C7B—C8B	−0.33 (14)	C2B—N3B—C12B—C11B	15.61 (11)
C6A—C7A—C8A—C8AA	0.29 (13)	C4B—N3B—C12B—C11B	−171.01 (7)
C6B—C7B—C8B—C8AB	0.15 (13)	C10B—C11B—C12B—N3B	−42.79 (10)

### Hydrogen-bond geometry (Å, º)

*Cg*3 and *Cg*9 are the centroids of the C5*A*–C8*A*/C4*AA*/C8*AA* and C5*B*–C8*B*/C4*AB*/C8*AB* rings, respectively.

**Table:**

*D*—H···*A*	*D*—H	H···*A*	*D*···*A*	*D*—H···*A*
N1*A*—H1*A*···Cl2	0.89 (2)	2.44 (2)	3.2659 (8)	155.9 (19)
N1*B*—H1*B*···Cl1^i^	0.83 (2)	2.352 (19)	3.1661 (7)	166.6 (18)
C11*A*—H11*A*···*Cg*9^ii^	0.99	2.67	3.5718 (10)	151
C12*B*—H12*D*···*Cg*3^iii^	0.99	2.57	3.4002 (10)	142

Symmetry codes: (i) −*x*+1/2, *y*−1/2, −*z*+1/2; (ii) −*x*+1, −*y*, −*z*; (iii) *x*+1/2, −*y*+1/2, *z*+1/2.
