# Supplementary material for: Crystal structure and Hirshfeld surface analysis of bis­(6,7,8,9-tetra­hydro-11H-pyrido[2,1-b]quinazolin-5-ium) tetra­chlorido­zincate
Source: Acta Crystallogr E Crystallogr Commun. 2021 May 14;77(Pt 6):629–33. doi: 10.1107/S2056989021004989 (PMC8183451; doi:10.1107/S2056989021004989)
Supplement: Supplementary file 3 [file e-77-00629-sup4.docx]

**Supporting Information**

***Table S1*** *Comparison for selected bond distances (Å) involved in the -N—C—N- moiety in the ring.*

| **Compounds** | | | **Bonds** | | | | | | | |
| --- | --- | --- | --- | --- | --- | --- | --- | --- | --- | --- |
|  |  |  | **residue A** | | | | **residue B** | | | |
|  |  |  | **N1A—C2A** | | **С2A—N3A** | | **N1B—C2B** | | **C2B—N3B** | |
| Title compound | protonated | 1.337 | | 1.310 | | 1.332 | | 1.311 | |  |
| EYUHEL | protonated | 1.333 | | 1.308 | | NA | | NA | |  |
| PYQAZP | protonated | 1.410 | | 1.386 | | 1.421 | | 1.520 | |  |
| GUCZUZ | unprotonated | 1.428 | | 1.471 | | NA | | NA | |  |
| LIZMOX | unprotonated | 1.458 | | 1.471 | | 1.463 | | 1.465 | |  |
